# Update on the therapeutic significance of estrogen receptor beta in malignant gliomas

**DOI:** 10.18632/oncotarget.20970

**Published:** 2017-09-18

**Authors:** Yu-Long Lan, Shuang Zou, Xun Wang, Jia-Cheng Lou, Jin-Shan Xing, Min Yu, Bo Zhang

**Affiliations:** ^1^ Department of Neurosurgery, The Second Affiliated Hospital of Dalian Medical University, Dalian, 116023, China; ^2^ Department of Pharmacy, Dalian Medical University, Dalian, 116044, China; ^3^ Department of Physiology, Dalian Medical University, Dalian, 116044, China; ^4^ Department of Neurology, The First Affiliated Hospital of Dalian Medical University, Dalian, 116011, China; ^5^ Department of Neurology, The Third People's Hospital of Dalian, Non-Directly Affiliated Hospital of Dalian Medical University, Dalian, 116033, China

**Keywords:** estrogen receptor β, glioma, expression, therapy

## Abstract

Malignant glioma is the most fatal of the astrocytic lineage tumors despite therapeutic advances. Men have a higher glioma incidence than women, indicating that estrogen level differences between men and women may influence glioma pathogenesis. However, the mechanism underlying the anticancer effects of estrogen has not been fully clarified and is complicated by the presence of several distinct estrogen receptor types and the identification of a growing number of estrogen receptor splice variants. Specifically, it is generally accepted that estrogen receptor alpha (ERα) functions as a tumor promoter, while estrogen receptor beta (ERβ) functions as a tumor suppressor, and the role and therapeutic significance of ERβ signaling in gliomas remains elusive. Thus, a deeper analysis of ERβ could elucidate the role of estrogens in gender-related cancer incidence. ERβ has been found to be involved in complex interactions with malignant gliomas. In addition, the prognostic value of ERβ expression in glioma patients should not be ignored when considering translating experimental findings to clinical practice. More importantly, several potential drugs consisting of selective ERβ agonists have exhibited anti-glioma activities and could further extend the therapeutic potential of ERβ-selective agonists. Here, we review the literature to clarify the anti-glioma effect of ERβ. To clarify ERβ-mediated treatment effects in malignant gliomas, this review focuses on the potential mechanisms mediated by ERβ in the intracellular signaling events in glioma cells, the prognostic value of ERβ expression in glioma patients, and various ERβ agonists that could be potential drugs with anti-glioma activities.

## INTRODUCTION

Glioma is the most common primary malignant brain tumor and has a poor prognosis. Currently more efforts should be directed toward developing more efficacious and targeted therapeutic paradigms. A known gender bias exists in tumor development: women have a lower incidence than men, indicating protective effects of estrogen. Evidence from various sources indicates that endogenous estrogens could have beneficial effects against glioma development [1]. First, the descriptive epidemiology of brain gliomas suggests a higher incidence in males than in females, particularly during premenopausal years [2]. Using New York State tumor registry data, McKinley et al. [2] calculated the incidence rates of age- and sex-specific for three types of glial tumors: anaplastic astrocytoma, glioblastoma multiforme (GBM), and astrocytoma not otherwise specified. The authors calculated the rate ratio for GBM in women relative to men based on 5-year age intervals and demonstrated that men are approximately 1.5–2 times more likely to develop proportional GBM. Besides, the rate ratio for GBM in women compared with men continued to decrease throughout the premenopausal years. These findings could suggest a protective effect of estrogens against glioma, especially during the premenopausal years. Second, some gliomas express various estrogen receptors (ERs) as well as aromatase (an important enzyme for the conversion of testosterone to estradiol) [1]. Third, experimental studies have revealed that glioblastomas transplanted into animals showed a slower growth rate in females than in males [1]. Furthermore, estrogen increased survival in an orthotopic model of glioblastoma [3]. Elucidating hormonal pathways that are found to be potentially involved in gliomagenesis could help design tumor-preventive strategies. In addition, since steroid hormones could penetrate the blood-brain barrier (BBB), it may be necessary to examine the relationship between circulating steroid levels and the subsequent risk of glioma [4]. Further epidemiological, experimental, and clinical studies are necessary to elucidate the effects of estrogen and estrogen compounds on the growth and proliferation of malignant gliomas.

As a steroid hormone in humans, the biological function of estrogen is primarily mediated by binding to two classical receptors: estrogen receptor alpha (ERα) and estrogen receptor beta (ERβ), which were identified and cloned in 1986 and 1996, respectively [5]. Our understanding of the role of estrogen in various tissues and the role of these two estrogen receptor subtypes has greatly improved over the past twenty years [6]. Because the expression of putative G protein coupled receptors (GPR30) has not been studied in gliomas, it is not discussed in this article. ERs can combine estradiol and other estrogen compounds to form homodimers and/or heterodimers. These dimers can also bind to a specific DNA sequence found in the regulatory regions of estrogen-responsive genes, called the estrogen response element (ERE), and recruit other components of the transcriptional machinery, thereby affecting gene expression [6, 7]. A number of coactivators and corepressors can modulate receptor function [7]. Furthermore, estradiol and other steroid hormones as well as selective estrogen receptor modulators (SERMs) can act through non-classical pathways (i.e., not directly involving ERs binding to EREs) [6, 7].

Although ERα and ERβ are structurally similar, their ligand binding domains actually exist sufficient differences to allow them to be selective for different ligands [8]. Recent studies have indicated that ERβ has a markedly different function than ERα [9] and is generally considered to be a tumor suppressor. Although ERα is detected in approximately 1/3 of all low-grade tumors [10], it has been reported that most gliomas are ERα negative [5]. ERα plays more prominent roles in the uterus and mammary gland, regulating metabolism, and preserving bone homeostasis, while ERβ could be of more pronounced effects on the central nervous system (CNS) [11]. Currently, the precise function, regulation, and mechanisms of action of ERα in human glioma are unknown. However, it is possible that ERα is reduced or absent during tumor development. Different from ERα, ERβ is expressed in glial neoplasms [12] and in non-neoplastic astrocytes [5]. Batistatou et al. [5] reported that the expression of ERβ was decreased in high-grade tumor tissues in parallel with their loss of differentiation. In other cancers, including prostate, breast, and ovarian cancers, the decrease or absence of ERβ is associated with a malignant phenotype, indicating that ERβ has a potential inhibitory effect on cancer [13–16]. In addition, it has been demonstrated that ERβ overexpression reduces cell proliferation whereas ERβ knockdown enhances cell proliferation in breast and colon cancer cells [17–19]. Although these studies suggest that ERβ has tumor-suppressive potential in some tumors, the role and therapeutic significance of ERβ signaling in gliomas remains elusive.

Given these research findings regarding the role of ERβ in preventing glioma, the protective effects of estrogen signaling cannot be ignored. The status and exact role of estrogen and ERs in glioma requires further research, and here, we review the literature regarding the roles of ERβ in malignant gliomas (Table 1). A thorough study of the role of ERβ in the genesis and development of diseases of the central nervous system (CNS) holds important clinical significance and could serve as a guide for the treatment of malignant glioma. Therefore, it is critically important to investigate ERβ with respect to the mechanism of its signal transduction pathway and, particularly, its correlation with glioma incidence.

**Table 1:** Published studies investigating the roles of ERβ in malignant gliomas

Series	Year	Journal	Country	Results
Batistatou A, et al.	2004	Journal of Cancer Research and Clinical Oncology	Greece	ERβ is mainly expressed in astrocytes of low-grade gliomas and in normal astrocytes. Its presence decreases with increased malignancy of these tumors.
Batistatou A, et al.	2006	Journal of Neuro-Oncology	Greece	ERβ is found to be expressed in gliomas and oligodendrogliomas. ERβ expression tends to decrease with increased histological malignancy of the tumor. Patients with ERβ-positive tumors could be of better prognosis and longer survival times.
Kim J,H et al.	2011	BMB Reports	Korea	ERβ promoted up-regulation of Egr-1 expression via a non-genomic mechanism involving the Raf/MEK1/Erk/Elk-1 signaling cascade.
Kefalopoulou Z, et al.	2012	Journal of Neuro-Oncology	Greece	ERβ and ER co-activators AIB1, TIF2, and PELP1 appear to play an important role in the pathogenesis and progression of astrocytic tumors and might have prognostic significance. The mechanisms underlying their involvement in astrocytic tumorigenesis, as well as their utility for prognostic and therapeutic purposes merit further investigation.
Sareddy G,R et al.	2012	Molecular Cancer Therapeutics	USA	ERβ signaling has a tumor-suppressive function in gliomas. Because ERβ agonists are currently in clinical trials and are well tolerated with fewer side effects, identification of an ERβ agonist as a therapeutic agent can be readily extended to clinical use with current chemotherapies.
Li W, et al.	2013	Brain Research	USA	ERβ5 is the main ERβ isoform found in gliomas. Hypoxia induced ERβ5 expression in glioma as a self-protective mechanism against tumor proliferation and that ERβ5 might serve as a therapeutic target for the treatment of glioma.
Liu C, et al.	2014	Cancer Epidemiology	China	This study indicated a combination of decreased expression of ERs, including ERβ, may be involved in the tumorigenesis of gliomas.
Liu X, et al.	2015	Molecular Medicine Reports	China	enhanced ERβ expression and sensitized glioma cells to TMZ-induced proliferation inhibition via the PI3K/AKT/mTOR pathway.

Abbreviations: TMZ: temozolomide.

## ERβ HAS COMPLEX INTERACTIONS WITH MALIGNANT GLIOMAS

**Figure 1:**
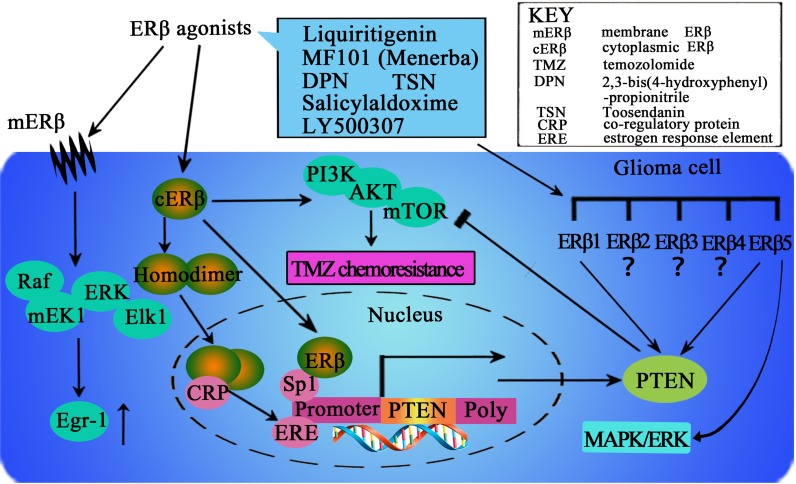
The potential mechanisms mediated by ERβ in the intracellular signaling events in glioma cells Increasing ERβ protein expression and nuclear translocation could be important for activation of the ERβ pathway. In addition, ERβ could stimulate Egr-1 transcription via the MEK1/Erk/Elk-1 cascade in glioma cells. Furthermore, ERβ agonist could enhance temozolomide sensitivity of glioma cells by inhibiting the PI3K/AKT/mTOR pathway. Ligand binding with ERβ could induce conformational changes that facilitate receptor dimerization to homodimers (ERβ/ERβ), translocation of dimers to the nucleus, and binding with co-regulatory proteins (CRP). This supra-molecular assembly thus may interact with the estrogen response element (ERE) in the promoter region of target genes. Furthermore, ERβ could also bind to the promoter region of PTEN through Sp1 and increase PTEN transcription. The presence of various ERβ isoforms could add further complexity to the action of ERβ, which should be considered in clarifying the effects of ERβ in human glioma. However, it remains unclear which isoforms primarily exert anticancer effects in human glioma, and the distinct function of each ERβ isoform is unknown. More efforts should be directed toward clarifying the activation and agonist-induced dynamics of ERβ/ERβ homodimers and the distinct function of each ERβ isoform to design more potent ligands that selectively activate ERβ.

Significant research efforts have been directed toward clarifying the relationship between malignant glioma and ERβ and investigating nuclear ERβ and the cytoplasmic part of ERβ, which have been shown to significantly influence glioma. Dominguez et al. [20] showed that estradiol rapidly regulates membrane ERs levels in hypothalamic neurons, which suggests that estrogen can regulate its own membrane signaling. Because the effects of estrogens are mediated largely through canonical ERs, primarily ERα and ERβ, the alterations in ERs levels that are observed in various regions of the brain in patients with glioma may be relevant to cancer malignancy. Further research has indicated that changes in cellular ERβ-mediated signaling could compromise the neuroprotective effects of estrogen and thereby contribute to the clinical progression of malignant glioma. The action of ERβ is necessary for estrogen to exert its neuroprotective effect; therefore, it is critically important to clarify the signaling responses triggered by ERβ that are relevant for anti-glioma effects (Figure 1).

## INCREASING ERβ PROTEIN EXPRESSION AND NUCLEAR TRANSLOCATION MAY BE IMPORTANT FOR ACTIVATION OF THE ERβ PATHWAY

Sareddy et al. [21] have explored the use of beta agonists as a potential novel therapeutic option to inhibit glioma growth. First, they detected ERβ expression in six glioma model cell lines, whereas little or no ERα expression was detected. Immunohistochemical analysis of tumor tissues indicated the downregulation of ERβ expression in high-grade gliomas. Importantly, they found that the expression of ERβ was significantly higher in normal brain tissues and low-grade tumors than in high-grade tumors. ERβ expression was decreased during the progression of gliomas and high-grade gliomas expressed ERβ predominantly in the cytoplasm. In addition, ERβ agonist treatment can lead to a marked decrease in the proliferation of glioma cells. Furthermore, activation of the ERβ pathway via agonists has the potential to increase ERβ protein expression and nuclear translocation. These findings indicated that ERβ agonists can promote the expression of ERβ and promote the tumor suppressive function of ERβ in glioma cells by increasing ERβ expression and nuclear translocation.

The findings of Sareddy et al. [21] suggested that ERβ-selective agonists such as DPN, MF101, and liquiritigenin have the potential to inhibit glioma cell proliferation and tumor growth. Thus, increased ERβ expression could be important for the effect of its agonists. Various ERβ-selective drugs including DPN, ERB-041, MF101, and liquiritigenin are currently being investigated as a replacement for estrogens to treat menopausal symptoms [8, 9]. Furthermore, these ERβ agonists (DPN and liquiritigenin) have good BBB permeability and exert less neuronal toxicity [22, 23]; hence, they are very suitable for the therapeutic treatment of gliomas.

## ERβ STIMULATES EGR-1 TRANSCRIPTION THROUGH THE MEK1/ERK/ELK-1 CASCADE IN GLIOMA CELLS

The Egr-1 gene is an immediate early response gene that encodes a transcription factor that plays a regulatory role in cell growth, differentiation, and apoptosis. Kim et al. [24] investigated whether ERβ could induce Egr-1 expression in glioma cells that express ERβ but not ERα. Their results showed that ERβ increased the expression of Egr-1 through a non-genomic mechanism involving the Raf/MEK1/Erk/Elk-1 signaling pathway.

ERs, including ERα and ERβ, are traditionally considered transcription factors that bind to EREs in the promoter region, resulting in the regulation of gene expression [24]. However, a non-transcriptional mechanism of signal transduction via ERα, the so-called non-genomic pathway, has been identified. Like ERα, ERβ at the cell membrane could also activate the non-genomic signaling pathway. In this investigation, the authors demonstrated that Erk1/2, rather than JNK1/2 or p38 MAPK, was promptly activated following 17β-E2 treatment in C6 glioma cells, which only express ERβ. Furthermore, pretreatment with the MEK1 inhibitor U0126 and the expression of dn mutants of MEK1, Erk2, or Raf1, blocked Egr-1 expression by ERβ. Thus similar to ERα, ERβ induces Egr-1 expressionvia activation of the Raf/MEK1/ERK/ELK-1 signaling pathway.

## ERβ AGONIST ENHANCES TEMOZOLOMIDE SENSITIVITY OF GLIOMA CELLS BY INHIBITING THE PI3K/AKT/MTOR PATHWAY

Temozolomide (TMZ) is a common first-line postsurgical drug used for malignant gliomas. However, the efficacy of TMZ is still poor because genetic resistance could be often observed. As mentioned above, ERβ has become an important tumor suppressor, as well as a key regulator of signal transduction in glioma cells; however, little is known regarding the role of ERβ in the chemotherapeutic response to TMZ. It is well known that PI3K/AKT/mTOR signaling plays a critical role in regulating protein synthesis, proliferation and survival and has been implicated in multiple drug resistance (MDR) in a variety of cancer types. Sveral key components of this pathway, including PI3K, AKT, epidermal growth factor receptor (EGFR) and Ras, have been observed to be frequently mutated in most tumor cells and are significantly related to the hyperactivation of this pathway [25]. Independent of O-6-methylguanine-DNA methyltransferase (MGMT, a DNA repair protein encoded by the MGMT gene that can remove cytotoxic and mutagenic adducts from the O^6^-guanine of DNA) function, the PI3K/AKT/mTOR signaling pathway may help to promote the expression of a series of anti-apoptotic factors and activate survival signals, which rid the cells of the cytotoxic effects caused by TMZ treatment [25, 26].

Using the novel highly selective ERβ agonist liquiritigenin and TMZ-resistant U138 glioma cells, Liu et al. [27] suggested that liquiritigenin treatment could enhance glioma cell susceptibility to TMZ through inhibiting the PI3K/AKT/mTOR pathway. They also suggested that function of liquiritigenin is dependent on ERβ and may be counterbalanced by IGF-1, which is a PI3K/AKT/mTOR activator. These results revealed a new MGMT-dependent mechanism of TMZ resistance, and emphasized the clinical use of liquiritigenin for improving the efficacy of TMZ. Because excessive activation of the PI3K/AKT/mTOR pathway is common in gliomas, the combined use of ERβ agonists may be a viable and effective treatment option to combat TMZ chemoresistance.

TMZ treatment in conjunction with liquiritigenin has been demonstrated to be a feasible therapeutic option with several benefits [27]. First, liquiritigenin was observed to be well tolerated, with almost no neuronal toxicity in phase II and III clinical trials [47, 48]. Second, liquiritigenin possesses strong BBB permeability capability and is able to reach the glioma cells [22, 23]. Third, ERβ agonists can selectively target cells expressing ERβ, making them more specific than other PI3K/mTOR inhibitors. Finally, the authors suggested that in addition to its role in decreasing TMZ resistance, liquiritigenin may also increase ERβ expression and inhibit tumor proliferation by activating the function of this well-known tumor suppressor [27]. These findings indicate that treatment with ERβ agonists may be a promising therapy for overcoming TMZ chemoresistance in human malignant glioma cells.

## THE PRESENCE OF VARIOUS ERβ ISOFORMS ADDS FURTHER COMPLEXITY TO THE ACTION OF ERβ THAT SHOULD BE CONSIDERED IN CLARIFYING THE EFFECTS OF ERβ IN HUMAN GLIOMA

ERβ has been found to be a potential tumor suppressor as mentioned above. Further identification of various ERβ isoforms further increases the complexity regarding the biomedical role of ERβ [28]. At present, at least 5 different ERβ isoforms have been identified, which have identical N-terminal sequences but diverge from amino acid 469 to the C-terminus [28]. *In vitro* analyses have revealed that each ERβ isoform has distinct transcriptional activity [28, 29]. The expression levels and functions of the different ERβ isoforms in breast cancer have also been studied and clarified [30, 31, 32]. Most studies on the expression of ERβ in cancer have used antibodies that do not distinguish between different ERβ isoforms, and much of the research on the function of ERβ in cancer has focused on the subtype of ERβ1. Various recent studies have indicated the great significance of aberrant ERβ expression in human glioma, its decreasing expression in high-grade tumors, and the inhibitory effect of ERβ agonist on the proliferation of glioblastoma cell lines [12, 21]. However, only immunohistochemistry was used to evaluate ERβ expression in these studies. At present, the expression of these isoforms in human gliomas is unclear, and the specific function of each ERβ subtype is also unknown for the time being.

Li et al. [33] have evaluated the expression of ERβ isoforms in human glioma using immunohistochemistry, real-time PCR, and Western blotting. In their study, they identified that ERβ5 is the predominant isoform of ERβ in human glioma. They found that over-expression of either ERβ1 or ERβ5 increased PTEN expression and, thus, inhibited activation of the PI3K/AKT/mTOR pathway. As ERβ over-expression increases PTEN levels in a ligand-independent manner [34], it could be speculated that upregulation of PTEN protein expression is a common anti-tumor mechanism of ERβ; however, this experiment was performed using 293T cells rather than glioma cells. Nonetheless, an in-frame deletion causes U87 glioma cells to be devoid of functional PTEN [35]. Further studies using glioma cell lines without a PTEN mutation are necessary to further support this prediction. The C-terminal ligand-binding domain (LBD) is considered to be the key structural domain of ERβ, controlling ligand-dependent regulation of the ER signaling pathway. Ligand binding in the LBD could induce conformational changes that facilitate receptor dimerization to form homodimers (ERβ/ERβ), translocation of dimers to the nucleus, and further binding with co-regulatory proteins [56]. This supra-molecular assembly finally interacts to the ERE in the promoter region of target genes [56]. In particular, the activation and agonist-induced dynamics of ERβ/ERβ homodimer need to be explored to design more potent ligands that selectively activate the ERββ homodimer. In addition, a recent study showed that ERβ can also increase transcription and expression of PTEN by binding the promoter region of PTEN via Sp1 [36]. The inhibitory effect of ERβ1 and ERβ5 on glioma cell proliferation identified currently also supports PTEN-independent mechanisms. In addition, the authors found that ERβ5 could inhibit the MAPK/ERK pathway. Their findings indicated that hypoxia-induced ERβ5 expression in gliomas may be a self-protective mechanism against tumor proliferation, and ERβ5 could be the therapeutic target for glioma treatment.

## THE PROGNOSTIC VALUE OF ERβ EXPRESSION IN GLIOMA PATIENTS COULD BE TRANSLATED INTO CLINICAL PRACTICE

Batistatou et al. [5] for the first time monitored ERβ expression immunohistochemically in 56 cases of astrocytomas of all grades (grade I-IV) and in adjacent non-neoplastic brain tissue. The authors demonstrated that moderate or strong nuclear immunopositivity was obtained in non-neoplastic astrocytes and in low-grade astrocytomas, whereas the majority of high-grade tumors were immunonegative or displayed weak immunoreactivity [5]. Intriguingly, there was a progressive decline in ERβ expression that paralleled the increase in tumor grade.

Interestingly, Batistatou et al. [12] further demonstrated that low ERβ expression was significantly correlated with high-grade tumors and worse survival in patients with astrocytic tumors. Multivariate analysis showed that ERβ expression had a prognostic value for overall survival in these patients [12]. Their results strengthen the hypothesis that ERβ could be of important role in the pathogenesis and progression of glial neoplasms [12]. In addition, ERβ protein expression could be a useful marker for identifying patients with better prognosis. The authors demonstrated that ERβ expression could be used as an independent prognostic marker for overall survival in patients with astrocytic tumors. To our knowledge, this is the first study to suggest that ERβ is important for prognostic evaluation in patients with astrocytic tumors. Although the sample size of this study was relatively small, the findings strongly favor a predictive effect of ERβ expression in the mortality of patients with astrocytic tumors. More research is needed to confirm these results, and further studies are needed to identify whether treatment strategies targeting ERβ are beneficial in gliomas.

Furthermore, Kefalopoulou et al. [37] for the first time explored the expression of ERβ pathway components in astrocytic tumors of different grades via an integrated approach and associated the tumors with patient prognosis and clinicopathological parameters. The authors demonstrated that the protein levels of ERβ and its co-activators, TIF2, AIB1 and PELP1, fluctuate as tumor grade increases in astrocytomas and are correlated with clinical and histopathological parameters [37]. Statistical analysis further indicated that ERβ expression in grade II astrocytomas was significantly higher than in grade III and grade IV astrocytomas. However, ERβ expression did not significantly differ between grade III and grade IV astrocytomas. These findings were further confirmed by Liu et al. [38], who demonstrated significantly higher levels of ERβ in low grade tumors (I and II) than in high-grade tumors (III and IV). In addition, univariate analysis indicated that in the total study population, low ERβ expression and high TIF2, AIB1, and PELP1 expression were associated with decreased overall survival. However, only low ERβ expression was predictive of shorter overall survival in a subgroup analysis stratified by grade [38]. Finally, multivariate Cox analysis that included age, grade, gender, and ERβ, TIF2, AIB1, and PELP1 expression revealed that high ERβ expression along with lower tumor grade were independent favorable prognostic factors of overall survival.

In summary, the expression of ERβ decreased with increased astrocytoma grade and could be a good independent prognostic factor of patient prognosis. Unveiling the complex mechanism of tumor initiation and progression is a necessary condition for optimizing tumor diagnosis and evaluation and to design new treatment strategies that are tailored and individualized. Further studies at the clinical and molecular levels are needed to confirm the current findings and to examine whether these findings can be translated to clinical practice.

## VARIOUS ERβ AGONISTS COULD EXERT ANTI-GLIOMA ACTIVITIES

ERβ-selective agonists are considered to be potential therapeutic agents for a wide variety of cancers [39–43]. Their development could be particularly challenging because differences in the ligand binding cavities of the two ER subtypes α and β are minimal. ERα plays a more prominent role in the mammary gland and the uterus, preserving bone homeostasis, and regulating metabolism. ERβ has more pronounced effects on the CNS and the immune system. Moreover, the β-subtype generally counteracts the ERα-promoted cell hyperproliferation in tissues such as breast and uterus and is generally considered a tumor suppressor in these organs. This antiproliferative effect of ERβ has also been found in several cancer tissues, such as breast [39], prostate [40], colon [41], renal [42], pleural mesothelioma [43], and glioma [21]. Particularly, the protective effect of ERβ in gliomas is also supported by the fact that the incidence rate of this cancer is smaller in women than in men [44], and more importantly, the use of exogenous estrogens further reduces this incidence [45]. All this evidence suggests that selective activation of this receptor subtype may be exploited to obtain an antitumor effect. Here, we review the ERβ-selective compounds that have been reported to be effective against malignant gliomas (Table 2).

**Table 2:** Summary of ERβ-selective compounds that have been reported to be of treatment effect on malignant gliomas

Compound	Origin	Chemical structure	Most recent results	References
Liquiritigenin	Glycyrrhiza uralensis	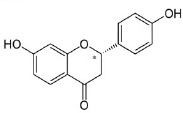	Liquiritigenin has the potential to inhibit glioma cell proliferation *in vitro* and also *in vivo* in xenograft-based assays.	Sareddy et al. (2012)
DPN	Synthetic	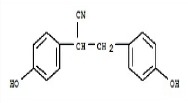	Treatment of glioma cells with DPN resulted in a significant dose-dependent reduction in cell proliferation	Sareddy et al. (2012)
Monoaryl-substituted salicylaldoxime	Synthetic	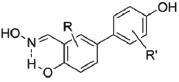	These deries of compounds were found to inhibit glioma growth in vitro were proved to be active in an in vivo xenograft model of human glioma, thus demonstrating the high potential of this type of compounds against malignant gliomas.	Paterni et al. (2015)
TSN	Melia toosendan Sieb. et Zucc.	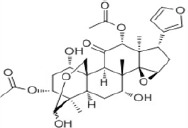	TSN is a candidate of novel anti-cancer drugs for malignant glioma and ER β and p53 were prominent targets for TSN.	Cao et al. (2016)
LY500307	Synthetic	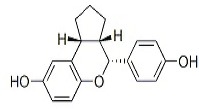	LY500307 treatment significantly reduced the in vivo tumor growth and promoted apoptosis of glioblastoma tumors in an orthotopic model and improved the overall survival of tumor-bearing mice.	Sareddy et al. (2016)

Abbreviations: DPN: 2,3-bis(4-hydroxyphenyl)-propionitrile; TSN: Toosendanin.

Recently, a great number of selective ERβ agonists have been developed and are being investigated and considered for therapeutic use [9]. Liquiritigenin, a novel highly selective ERβ agonist, was recently isolated from the Glycyrrhiza uralensis [46]. Liquiritigenin is an active compound found in MF101 (Menerba), a plant extract previously designed to treat vasomotor symptoms (hot flashes) associated with menopause [46]. In a phase II clinical trial of Menerba [47], the drug was found to be safe, well tolerated, and taken with high compliance. It is being further evaluated for its therapeutic use in a phase III clinical trial [48]. Sareddy et al. [21] investigated the status and significance of ERβ signaling in gliomas through the use of both *in vitro* and *in vivo* xenograft models of gliomas and tested its therapeutic significance using recently developed selective ERβ modulators. Their findings indicated that ERβ agonists could promote both the expression and tumor-suppressive functions of ERβ. They demonstrated that liquiritigenin significantly reduced *in vivo* tumor growth in a xenograft model [21]. Significantly, their results suggest that ERβ signaling plays a tumor-suppressive function in gliomas, and thus, ERβ agonists could represent a novel class of drugs for curbing glioma progression. In addition to the results described above, various other ERβ-selective drugs such as ERB-041, 3,3′-diindolylmethane (DIM) should also be considered when exploring the roles of ERβ agonists in malignant glioma treatment [8, 9], although no reports have been published regarding their treatment effect on glioma.

Paterni et al. [11] investigated the optimization of selective ERβ agonists that were developed and improved by structural refinements of a monoaryl-substituted salicylaldoxime scaffold [49–51]. Their study described how molecular modeling revealed a simple way to introduce molecular variations that produced some salicylketoxime derivatives with significantly improved binding affinity, transactivation activity, and subtype selectivity over their aldoxime counterparts. In addition, for the first time, further pharmacological evaluations were conducted on their oxime-based ERβ-agonists, both *in vitro*, on a glioma U87 cell line, and *in vivo* on a murine xenograft model of the same tumor.

As mentioned above, the plant-derived ERβ agonist liquiritigenin has been proven to suppress the growth of subcutaneous glioma xenograft tumors [21]. In addition, salicylketoxime-based ERβ agonists have also been found to reduce glioma growth in subcutaneous models [11]. Furthermore, the new potential ERβ agonist toosendanin (TSN) has also been shown to reduce tumor burden in a xenograft model of athymic nude mice [52]. However, these studies are limited by the lack of drug testing using an orthotopic model, and furthermore, when testing the efficacy of glioma chemotherapy drugs, the tumor microenvironment and the presence of a complete immune system must be considered. Thus, using both orthotopic tumor models and a syngeneic model with an intact immune system, Sareddy et al. [53] investigated the effect of LY500307. The authors demonstrated that the selective ERβ agonist LY500307 reduced glioma progression and enhanced survival in syngeneic mouse models. As LY500307 readily crosses the BBB, it is currently being tested in clinical trials and has been found to be well tolerated. Thus significantly, it can be readily transferred to current clinical chemotherapy, thus providing an additional adjunct to improving the survival time of patients with gliomas, with limited drug toxicity.

Several efforts have been directed towards the development of ER-selective ligands [54]. In particular, significant attention has been focused on ERβ-selective agonists [55], which have the potential to be used as antitumor agents because they predominantly activate the β-subtype and thus circumvent the undesired ERα-promoted proliferative effects on the breasts and the uterus. These compounds have been proven to act as full agonists of ERβ and to activate the transcription of reporter genes and endogenous genes, thus highlighting their extremely high ERβ selectivity. These results further extend the therapeutic potential of ERβ-selective agonists.

## CONCLUSIONS

This review focuses on the therapeutic significance of the ERβ pathway in gliomas and suggests that functional activation of the ERβ pathway may be a potential therapeutic target for gliomas. Although ERβ has been shown to have tumor-suppressive potential, more efforts should be directed toward clarifying the exact roles of ERβ in the etiology and progression of malignant glioma. Likewise, limited information regarding which promoters drive classical ER transcription in glia or which ERβ splice variants are expressed in glia and glial tumors is currently available. Further identification of certain genes that are specifically regulated by ERβ warrants further research. Because ERβ agonists are currently being tested in clinical trials and have been well tolerated with few side effects, the identification of ERβ agonists as therapeutic agents could be readily extended to clinical use, and thus, ERβ agonists could represent a novel class of drugs for treating gliomas in the near future.
